# Mitochondrial function is impaired in long COVID patients

**DOI:** 10.1080/07853890.2025.2528167

**Published:** 2025-08-12

**Authors:** Jane Macnaughtan, Kai-Yin Chau, Ewen Brennan, Marco Toffoli, Antonella Spinazzola, Toby Hillman, Melissa Heightman, Anthony Henry Vernon Schapira

**Affiliations:** a Department of Clinical and Movement Neurosciences, UCL Queen Square Institute of Neurology, University College London, London, United Kingdom; b Institute for Liver and Digestive Health, London, United Kingdom; c Department of Respiratory Medicine, University College London Hospitals NHS Foundation Trust, London, United Kingdom

**Keywords:** ATP synthase, long COVID, mitochondrial dysfunction, peripheral blood mononuclear cells (PBMCs)

## Abstract

**Background:**

The Long COVID syndrome is a major global health problem, affecting approximately 10–20% of individuals infected with SARS-CoV-2 virus with many remaining symptomatic beyond one year. Fatigue, reduced exercise tolerance and hyperlactataemia on minimal exertion have led to the suggestion of a bioenergetic defect. We hypothesised that mitochondrial dysfunction is a pathological feature in Long COVID cases and would correlate with clinical outcome.

**Methods:**

This prospective, case-controlled, observational study recruited 27 participants with an established diagnosis of Long COVID syndrome from a single tertiary clinic together with 16 age-matched controls aged 25–65 years. Seahorse-based mitochondrial flux analysis and bioenergetics profile of isolated peripheral blood mononuclear cells (PBMCs) was performed and correlated with clinical phenotype.

**Findings:**

Long COVID cases had an increased baseline and ATP-induced oxygen consumption rate with a significant attenuation in tetramethylrhodamine methyl ester perchlorate fluorescence response to oligomycin. Correlations were observed between mitochondrial function and autonomic health, quality of life and time from index infection. Sex-specific differences were also observed.

**Interpretation:**

PBMCs from Long COVID subjects exhibit an exceptional and distinctive change in ATP synthase, as it contributes to the mitochondrial membrane potential rather than using it exclusively to generate ATP. The findings suggest that the enzyme runs both forward and reverse reactions, synthesising and hydrolysing ATP. The correlation of mitochondrial function with clinical phenotype in Long COVID may indicate a causal relationship and warrants further validation in larger scale studies.

## Graphical abstract

**Figure:**
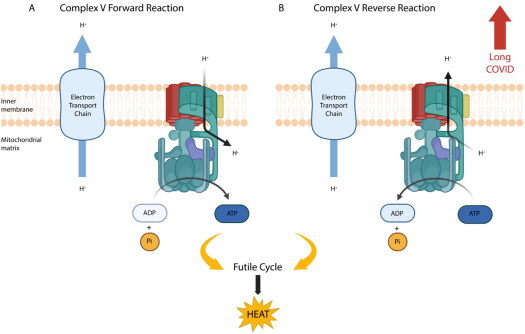

## Introduction

The global SARS-CoV-2 (COVID-19) pandemic that began in late 2019, caused more than 187 million infections and 4 million deaths by July 10, 2021 [1]. A substantial proportion of survivors experience long-lasting medical and psychological disability with significant economic consequences [2]. According to the World Health Organization definition, Long COVID (LC), occurs in individuals with a history of probable or confirmed COVID-19 infection, usually 3 months from the onset of infection with symptoms that last for at least 2 months and cannot be explained by an alternative diagnosis [3]. LC syndrome is recognised to be a consequence of acute infection, but its severity is not related to that of the initial illness and may occur in those hospitalised or not hospitalised with their infection [4–6]. The median proportion of survivors experiencing at least one persistent post-acute sequela of COVID-19 at one month has been shown to be 54.0% (45.0–69.0%); at 2–5 months, 55.0% (34.8–65.5%); and at 6 or more months, 54.0% (31.0–67.0%) [7]. The most common symptoms include difficulty concentrating, memory deficits, cognitive impairment, reduced mobility, fatigue, and muscle weakness.

The SARS–CoV-2 virus is able to evade pathogen recognition receptors by inducing the production of double membrane vesicles with the aid of mitochondria and the endoplasmic reticulum [8]. Abnormal mitochondrial function has been described in peripheral blood mononuclear cells (PBMCs) in cases with acute COVID-19 infection, including reduced oxygen consumption rate, mitochondrial depolarisation, a shift to glycolysis and increased mitochondrial mass with abnormal morphology [9–11]. Increased levels of circulating mitochondrial DNA have also been identified in those with acute COVID-19 infection [12,13] being these levels associated with worse outcome [13]. Most recently, acute COVID-19 infection has been reported to block transcription of a subset of nuclear-encoded respiratory chain proteins, induced glycolysis and activated host immune defences in a mouse model and human autopsy samples [14].

The clinical features of LC with the development of a lactic acidosis are reminiscent of defects of mitochondrial oxidative phosphorylation [15] characterised by chronic muscle weakness and fatigue and are often accompanied by a range of additional features of multisystem involvement and cognitive impairment [16]. Studies describing cases affected by COVID have demonstrated important differences between those at risk of severe acute COVID, and those with a diagnosis of LC. Cases with LC tend to be younger, are more likely to be female, and less likely to be from non-white ethnic heritage [6,17]. De Boer et al. observed a metabolic phenotype of decreased fat oxidation and increased lactate production per power output in a cohort of Long COVID cases [15], suggesting that these alterations could contribute the functional limitation in LC cases. However, little is known about the specific features of mitochondrial respiratory capacity that could contribute to the LC syndrome. To address this gap in knowledge we investigated the mitochondrial function of PBMCs of subjects with LC using metabolic and fluorescence-based approaches.

## Methods

### Study design and population

This investigator-led, single centre, prospective, observational study was conducted in the tertiary out-patient LC service at University College Hospital, London. 27 cases with a diagnosis of LC syndrome and 16 healthy age-matched controls were recruited between August 2021 and August 2022. Written and informed consent was obtained from all participants.

Case inclusion criteria were as follows: LC syndrome as defined by the WHO criteria (the continuation or development of new symptoms 3 months after the initial COVID-19 infection, with these symptoms lasting for at least 2 months with no other explanation) [3]; Age 25–65 years inclusive; Fatigue Assessment score of 22 or above. Case exclusion criteria were: active COVID-19 at the time of recruitment; a prior diagnosis of chronic fatigue syndrome; alcohol consumption within the previous week more than 14 units for women and 21 units for men. (Alcohol thresholds were pragmatically designed to exclude excessive alcohol consumption yet reflect the sex differences in alcohol metabolism). Healthy controls were recruited between the ages of 25–65 years. Exclusion criteria were: acute or suspected COVID-19 within the preceding 12 weeks; COVID-19 vaccination within the preceding 12 weeks. Cases and controls with a history of cancer within the previous 5 years and diabetes mellitus were excluded. The control group was selected to age-match the case group. All cases had an established diagnosis of LC as determined by the tertiary LC service. The healthy controls were a mixed population of individuals who had recovered from SARS-CoV-2 infection with no active clinically relevant disease and those naïve to the virus. Sub-group analysis was not conducted due to small sample size.

#### Clinical assessment of LC cases

Clinical information collected at the time of clinical assessment included the following: Fatigue Assessment Scale © FAS (Fatigue Assessment Scale), a well validated tool to assess both mental and physical fatigue [18,19]: ild care foundation (www.ildcare.nl); MRC Dyspnoea scale (used with the permission of the Medical Research Council); EQ5D-5L Quality of life score (Euroqol); Resting Heart Rate/Maximum Predicted Heart Rate as a measure of autonomic dysfunction [20]; presence of myalgia. These data we collected from the LC cases only. Data including age, sex and body mass index (BMI) were also collected from both cases and controls.

#### Peripheral blood mononuclear cell isolation

10**–**15 ml of whole blood was collected into Vacutainer EDTA tubes (Becton Dickinson, UK) and processed within 2 h. Lymphoprep was purchased from BioVision (UK) or STEMCELL Technologies (UK) and was used to isolate PBMCs according to the manufacturers’ instructions. PBMC were washed twice in Gibco’s 1x DPBS (ThermoFisher Scientific, UK) and viable PBMC were counted using Moxi GO using a size gate between 6 and 15um by following the manufacturer’s instructions [21].

### Mitochondrial studies

SeaHorse assay was conducted by following the manufacturer’s instructions and specifically optimized as described [22]. Analysis was performed using SeaHorse Xfe24 operated by Wave 2.6.4 version, Cytation-1 reader operated by Gene5 3.16.10 and FlowJo 10.10.0.

*Oxygen consumption measurements*: The XFe24 plate was first coated with Cell-Tak (Corning, USA). Typically, 800,000 PBMCs were seeded onto each well, in 500 µl of completed assay medium. The SeaHorse analyser (Agilent, UK) was programmed to inject sequentially oligomycin (to the final concentration of 1.5 µM), carbonyl cyanide-4 (trifluoromethoxy) phenylhydrazone (FCCP) (to the final of 1.5 µM) and antimycin and rotenone (to the final of 1 µM). Four technical repeats per participant were measured. Any outliners of over 3x SD were eliminated. Basal oxygen consumption rate (OCR) was calculated as the difference between the OCR before oligomycin injection and that after injecting antimycin and rotenone. Adenosine triphosphate (ATP)-linked OCR was calculated as the difference between the OCR before and after oligomycin injection. Proton-leak OCR was calculated as the difference between the OCR after injection of oligomycin, followed by antimycin and rotenone. Cells in the wells were digested by PrepGEM (Forenteg, UK) and DNA stained by Picogreen (ThermoFisher Scientific) and fluorescence measured in Cytation-1 reader (Agilent). OCR values were then normalised to picogreen signal.

*Mitochondrial content measurements:* Citrate synthase (CS) activities were measured as a marker of mitochondrial content, as described by Pryde et al. [23]. The relative CS activities were calculated as the percentage change of the sample comparing to a reference sample that is expressed as 100%. Mitochondrial content was also measured from the abundance of translocase of outer membrane (TOM)-20 by ELISA according to the manufacturer’s instructions (FineTest, China).

*Tetramethylrhodamine methyl ester (TMRM) staining*: 4 vials each containing 25,000 PBMC in 70 µl of Hanks’ Balanced Salt Solution (HBSS) were stained in 25 nM TMRM (ThermoFisher Scientific) and incubated at 37 °C for 15 min. Then oligomycin, FCCP, antimycin A and rotenone were added separately to a final concentration of 1.5 uM, 1.5 uM and 1 uM respectively and incubated for another 15 min. The stained cells were analysed by Moxi GO using the 561 nm filter and the medium gain. A fluorescent reference was also measured from the low intensity Nile Red fluorescent particles (5–7.9 μm; Spherotech, USA). Data were analysed by FlowJo (Becton Dickinson), first by gating on 10^2^ fluorescence and above and then 6 μm and above. Unstained controls were used for gating. The gated events were converted to a histogram and the mean fluorescent intensity (MFI) was obtained per condition, including the fluorescent reference (Figure 3D). TMRM fluorescence was expressed as the difference between the untreated and the antimycin A and rotenone treated, divided by the reference fluorescence. The % Δ TMRM upon treatments was produced by the percentage difference from the untreated.

**Figure 1.**
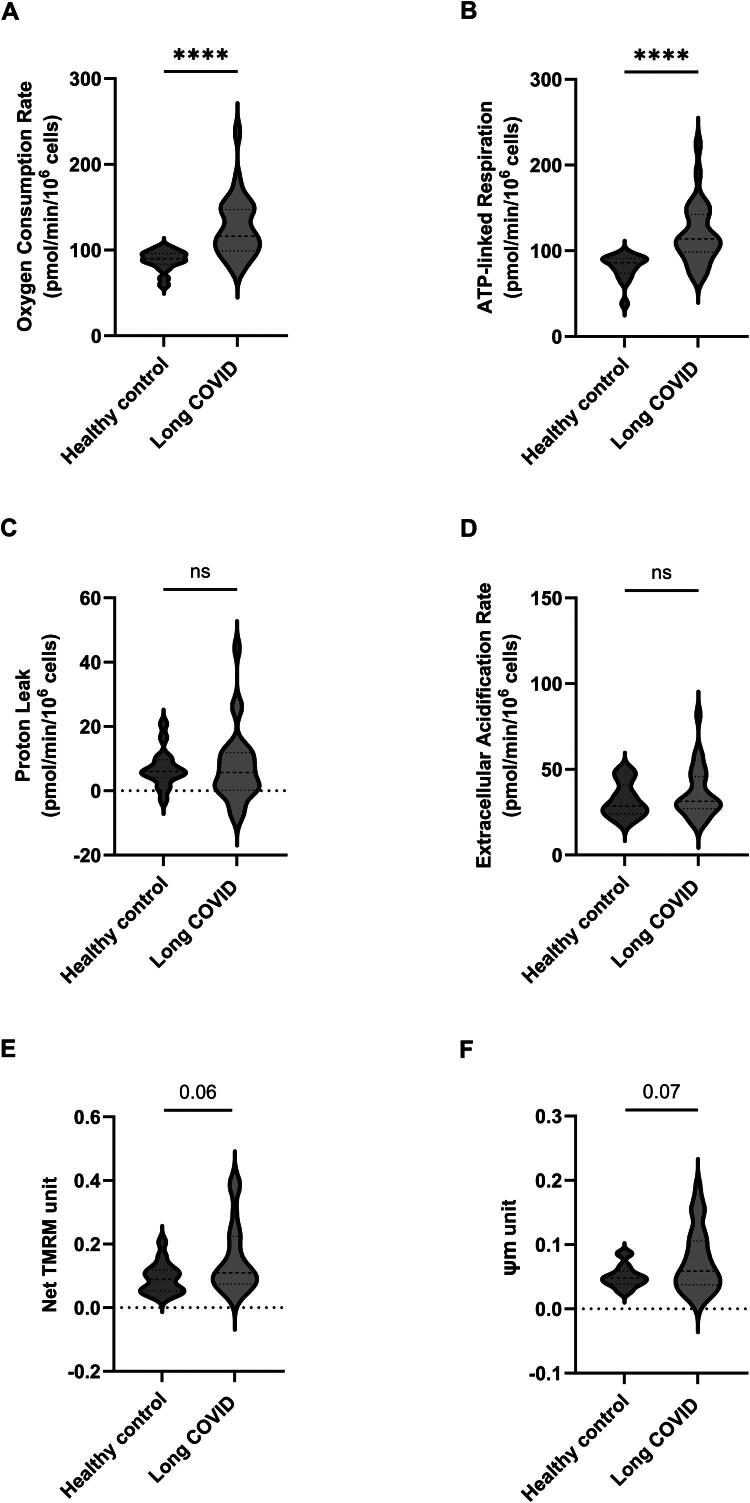
Comparison of oxygen consumption rate (OCR) of PBMCs collected from long-COVID cases (*n* = 27) and controls (n = 16). Measurements were performed by seahorse analyser and OCR was corrected by post-run cell number determination. (A) The basal OCR; (B) shows the ATP-linked OCR. (C) and (D) illustrate proton-leaked OCR and extracellular acidification rates (ECR) between the 2 groups, respectively. (E) and (F) represent tetramethylrhodamine methyl ester perchlorate (TMRM) staining and the mitochondrial membrane potential (ψm) respectively (data is expressed as median with interquartile range, and medians are compared using the Mann–Whitney *U* test) A.U. Arbitrary Units.

*Mitochondrial (mt) DNA measurements:* To the cells digested by Prep GEM, Cyclooxygenase-2, Amyloid Precursor Protein-1 abundance was measured as previously described [24] to quantify the ratio between mt/genomic (g)-DNA, using SYBR Green master mix and analysed by the StepOne qPCR machine (ThermoFisher). Long range polymerase chain reaction was conducted across the most common site of deletion, the major arc of mtDNA, between nucleotide 3984 and 110 to detect mtDNA rearrangement as previously described [24–26] (LA Taq DNA Polymerase (Takara Bio, France)).

#### Statistics

Discrete variables were described as counts (percentage) and continuous variables as mean (Standard Deviation (SD)). Non-normally distributed variables were described by the median (*Q*1–*Q*3). The Spearman’s rank correlation coefficient was calculated to describe associations between mitochondrial and clinical parameters. The Mann–Whitney *U* test was used for analysis of all continuous variables. In all statistical analyses, significance was set at *p* < 0.05. In our analysis, we considered *p* values between 0.05 and 0.1 as indicative of a statistical trend. Hodges–Lehmann (HL) estimates were calculated for non-parametric data. Categorical data was analysed using Chi Square analysis. As an exploratory study, power calculations were not determined, and cohort sizes were based on feasibility. The p values were not corrected for multiple comparisons due to the small size of the study. Post hoc stratification by sex was performed.

#### Ethical approval

Ethical approval for this study (Reference NC01.21) was obtained from the UCL/UCLH Biobank for Studying Health and Disease is licensed by the Human Tissue Authority (Ethics number: 20-YH-0088) in 2021. The study is registered to the UCL/UCLH Biobank for Studying Health and Disease which is listed on the Health Research Authority publicly available database. (https://www.hra.nhs.uk/planning-and-improving-research/application-summaries/research-summaries/ucluclh-biobank-for-studying-health-and-disease-renewal-2020/*)*

#### Role of funders

The study was supported by UCLH and the Royal Free Charities and funding for EB was awarded by the Howard de Walden Estate.

## Results

### Study population

A total of 27 cases and 16 controls were studied. All cases satisfied the WHO criteria for LC [3]. Median age values of control and cases were 36.5 and 42.0 years respectively (*p* = 0.19). A non-significant female preponderance was observed in the case cohort, reflecting the previously described demographic in this group. The case group had a significantly higher median BMI of 25.6 compared to controls vs 24.0 kg/m^2^ (*p* = 0.02) (Table 1).

**Table 1.** Case and control group characteristics with clinical parameters used in the assessment of long COVID syndrome; the Mann–Whitney test was performed on all continuous variables and expressed as median (25th–75th percentile values).

Clinical parameter	Healthy controls (*n* = 16)	Long-COVID (*n* = 27)	*p* value
Age (Median years, *Q*1–*Q*3)	36.5 (32.25–45.00)	42 (37.00–44.00)	0.19
Sex (*n* (%))			
Female	8 (50)	20 (74)	0.11
Male	8 (50)	7 (26)	
Ethnicity (*n* (%))			
White	13 (81.25)	20 (74.1)	0.59
Asian	2 (12.5)	2 (7.40)	
Black	1 (6.25)	3 (11.1)	
Arab	0 (0)	2 (7.40)	
Body mass index (Median, *Q*1–*Q*3)	24.0 (21.05–25.00)^a^	25.60 (22.80–32.51)	0.003
Clinical parameter		Median	*n*
Fatigue assessment scale	NA	34.00 (31.00–39.00)	23
MRC dyspnoea scale	NA	2.00 (1.00–3.00)	24
Resting HR/predicted maximum HR (%)	NA	45.00 (39.25–48.75)	24
EQ5D-5L	NA	11.00 (9.00–13.00)	23
Time from index COVID infection (days)	NA	535.00 (330.80–693.00)	24
Time from last vaccination (days)	NA	140.00 (46.50–281.00)	21
Myalgia (*n* (%))			
Yes	NA	13 (48)	
No	NA	14 (52)	
Hospitalised during index infection (n, (%))	NA	4 (15)	

Discrete variables were described as counts (percentage) and categorical variables by Chi-squared analysis. In all statistical analyses, significance was set at *p* < 0.05, NA - Not assessed..

^a^Data available for body mass index in 13 healthy controls and 27 long COVID patients.

The median duration since the index SARS–CoV-2 infection in the LC group was 535.0 days and the mean duration since last COVID-19 vaccination was 140.0 days. Four cases (15%) were admitted at the time of their index infection, but none required mechanical ventilation. Mild to moderate fatigue is defined as a Fatigue Assessment Scale score of 22–34 and severe fatigue 35 or above. The median value of the fatigue assessment score in the LC group was 34. The median MRC Dyspnoea score was 2, indicative of moderate dyspnoea on exertion. Median resting heart rate expressed as a percentage of maximum predicted heart rate was 45.0%. 48% of cases reported symptoms of myalgia. A summary of clinical parameters grouped by sex is described in Supplementary Table 2.

#### Mitochondrial function

**Figure 2.**
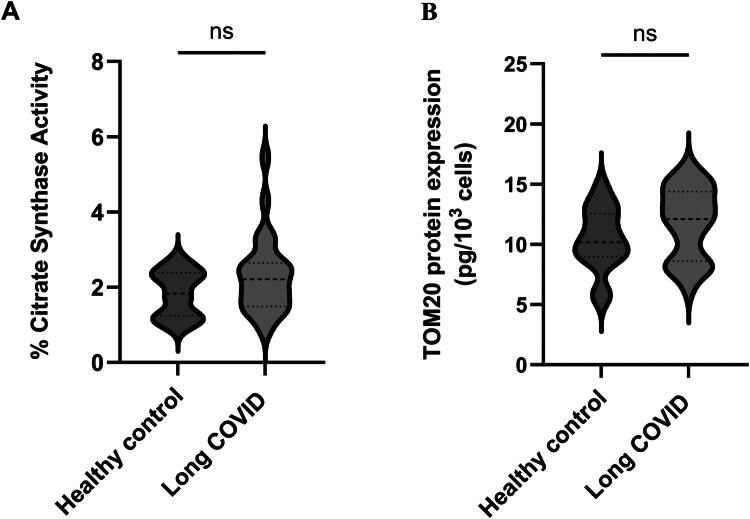
Comparison of mitochondrial biomass parameters of peripheral blood mononuclear cells from patients (*n* = 27) and controls (*n *= 16). Panels A and B, respectively, represent percentage citrate synthase activity and TOM20 protein expression (data is expressed as median with interquartile range, and medians are compared using the Mann–Whitney *U* test).

**Figure 3.**
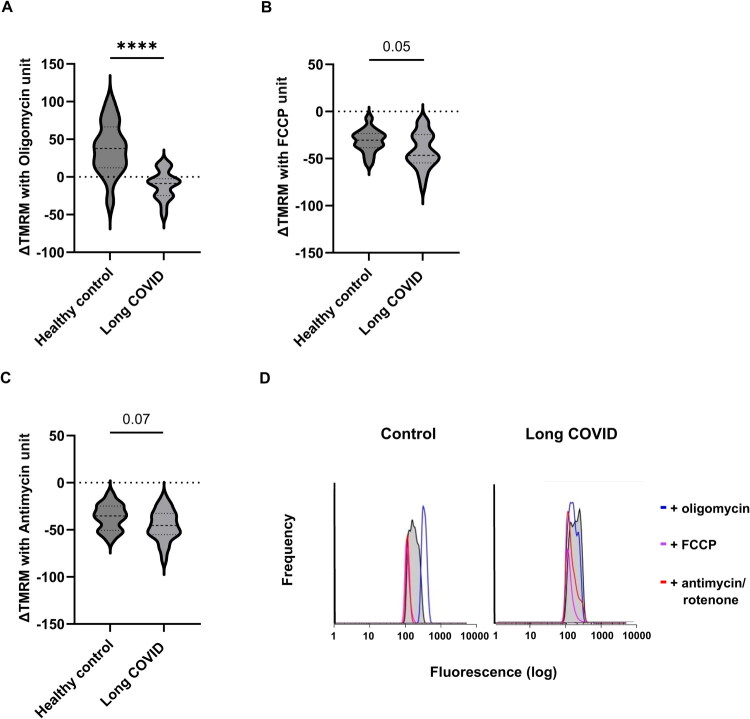
Comparison of tetramethylrhodamine methyl ester perchlorate (TMRM) dynamic of peripheral blood mononuclear cells collected from patients (*n* = 27) and controls (*n* = 16). Panel A–C show the change in TMRM upon addition of oligomycin, FCCP and antimycin/rotenone respectively. Panel D illustrates the changes in TMRM by histograms (data is expressed as median with interquartile range, and medians are compared using the Mann–Whitney *U* test).

The 27 cases displayed significantly higher basal respiration compared to the 16 controls, median OCRs of 116 and 90, respectively (HL 32.8) (*p* < 0.0001) [(Figure 1A, Supplementary Tables 1 and 3]. The increase was maintained when analysed by sex across groups, although the difference was greater in males than females (Supplementary Table 1). A similar significant increase in oligomycin-sensitive respiration (114 vs. 86) (HL 30.4) (*p* < 0.0001) (Figure 1B) was observed in cases compared to controls although there was no evident mitochondrial uncoupling as evidenced by a similar proton leak (Figure 1C). No difference in aerobic glycolysis (to lactate) was observed as evidenced by Extracellular Acidification Rate (ECAR) in cases and controls groups (Figure 1D). Net TMRM (Figure 1E) and membrane potential (Figure 1F) were not significantly different between groups. No significant difference in mitochondrial mass was observed as evidenced by comparable CS activity and TOM20 abundance between the 2 groups (Figures 2A and 2B). A significant difference in mitochondrial membrane polarisation in LC PBMC vs controls upon oligomycin treatment (Figures 3A and B, *p* < 0.00001). This indicates that the mitochondria in LC PBMCs have a diminished capacity to support the mitochondrial membrane potential when ATP synthase is inhibited. No significant differences in ΔTMRM were observed with FCCP or antimycin treatment (Figures 3B and C). No differences in mtDNA levels and damaged mtDNA molecules (deletions) were observed between groups (Supplementary Figure 1).

#### Mitochondrial function and clinical phenotype

Resting heart rate expressed as a percentage of maximum predicted heart rate was used as a measure of autonomic dysfunction. A significant positive correlation was observed with basal OCR (*R* = 0.42, [CI −0.008–0.703], *p* < 0.05) and negative correlation with membrane potential (*R* = –0.45 [CI −0.727 to −0.042], *p* < 0.05) suggestive of an association between mitochondrial integrity and autonomic function (Table 2). A trend to positive correlation with ATP-linked OCR was also observed (*R* = 0.38 [CI −0.043 to 0.685], *p* = 0.069). There was a significant positive correlation of quality-of-life scores as determined by EQ5D-5L with ECAR (*R* = 0.49 [CI 0.083–0.756], *p* < 0.05) and negatively with oligomycin-induced changes in TMRM (*R* = −0.45 [CI −0.732 to –0.031], *p* < 0.05). FCCP-induced changes in TMRM were negatively correlated with time from index infection (*R* = −0.43 [CI −0.716 to −0.019], *p* < 0.05). A trend for a negative correlation was observed between oligomycin-induced TMRM and time from index infection (*R* = −0.40 [CI −0.695 to 0.023], *p* = 0.056).

**Table 2.** Correlation analysis of mitochondrial function against clinical features of long COVID syndrome by spearman rank analysis with associated correlation matrix demonstrating Spearman *R* values; statistical significance was set at *p* < 0.05.

Variables	Spearman *R*	95% CI	*p* value
HR/Max HR vs Basal OCR	0.407	−0.0078 to 0.7029	0.048
HR/Max HR vs ATP-linked OCR	0.378	−0.0427 to 0.6847	0.069
HR/Max HR vs ψm	−0.448	−0.7271 to −0.0417	0.028
EQ5D-5L vs ECR	0.489	0.0834 to 0.7557	0.018
EQ5D-5L vs oligomycin-induced TMRM	−0.448	−0.7324 to −0.0314	0.032
Time from index infection vs FCCP-induced TMRM	−0.430	−0.7162 to −0.0190	0.036
Time from index infection vs oligomycin-induced TMRM	−0.395	−0.6951 to 0.0229	0.056

#### Mitochondrial function stratified by sex

Post hoc stratification of mitochondrial function by sex revealed significant reductions in FCCP- (LC −46.53 vs HC −25.31, *p* < 0.05) and antimycin A/rotenone TMRM (LC −48.38 vs HC −31.87, *p* < 0.01) in female but not male cases compared to controls (Supplementary Table 1). Significantly higher net TMRM staining was observed in female cases compared to female controls (LC 0.12 vs HC 0.05, *p* < 0.01) (Supplementary Table 1). Sex differences in the mitochondrial phenotype of long COVID were observed. Males were characterised by more pronounced difference in oxygen consumption rates compared to controls. Females in contrast exhibited more striking differences in markers of membrane polarisation.

## Discussion

The Long COVID syndrome is a major cause of morbidity worldwide with a symptom complex highly suggestive of an underlying bioenergetic defect. It is a distinct clinical entity from acute SARS–CoV-2 infection both temporally and in relation to symptomatology, with a clear female preponderance [19]. Whilst multiple studies have described mitochondrial dysfunction in the context of acute SARS–CoV2 infection, few studies have evaluated the role of mitochondrial function in LC and none, to our knowledge, have assessed oxidative phosphorylation via flux analysis in the PBMCs of LC cases. This study evaluated live measures of mitochondrial function in the PBMCs from an out-patient case cohort with LC and age-matched controls.

### PBMC mitochondrial function in Long COVID is distinct from that of acute SARS–CoV-2 infection

Previous studies have reported abnormal mitochondrial function in the PBMCs of individuals with acute SARS–CoV-2 infection, including reduced oxygen consumption [27], mitochondrial depolarisation [28], a shift to glycolysis [11,29], increased mitochondrial mass [30] with abnormal morphology [31] and increased circulating mitochondrial DNA concentrations [30,32,33]. This metabolic ‘rewiring’ represents a subversion of host cellular metabolism by SARS–CoV-2 to facilitate viral replication and immune host evasion with selective targeting of mitochondria by viral proteins [34]. In contrast, the findings from this study suggest that in LC, PBMCs display no changes in glycolysis, mitochondrial mass or mitochondrial depolarization. Instead, LC-PBMCs exhibit higher rates of oxidative phosphorylation and ATP synthase activity compared to control with complex V observed to contribute to the maintenance of the mitochondrial membrane potential. This observation in LC PBMCs is perhaps more readily unmasked because chronic phases of inflammation are more reliant on oxidative phosphorylation in contrast to glycolysis, which is the preferred energy source in the acute phase of inflammation [35].

### PBMC complex V activity is aberrant in long COVID

Ordinarily, ATP synthase utilizes the proton gradient generated by the respiratory chain to make the majority of the cell’s ATP; as such, its activity decreases the mitochondrial membrane potential. In contrast, in LC PBMCs, complex V *contributes* to the proton gradient, as the membrane potential decreases when ATP synthase is inhibited with oligomycin. This phenomenon is well understood, as the enzyme can run in reverse, hydrolysing ATP and translocating protons across the inner mitochondrial membrane *via* the c-ring (see graphical abstract). While increased reverse ATP synthase can occur in respiratory chain deficiencies, there appear to be no such requirement in LC PMBCs, as the respiratory chain is more, not less, active than normal and there is no excessive proton leak; thus, there is no apparent physiological need for ATP synthase to contribute to the mitochondrial membrane potential in LC PBMC. Given that the membrane potential is not appreciably higher in LC PBMCs than control, the opposing activities of ATP synthase, ATP synthesis and hydrolysis, must cancel each other out, creating a futile cycle that will dissipates energy as heat. We tentatively propose that this is an anti-viral response mechanism (subcellular ‘fever’ response) that serves to shift the metabolic balance from anabolism to catabolism. Such a response is potentially helpful in the acute infection stage, by counteracting the aforementioned changes that ‘subvert’ cellular metabolism for viral production. However, in LC, this catabolic enhancement appears to continue long after viral infection, despite it no longer serving a useful purpose, and this could be a cause of fatigue and quality of life measures. Indeed, the significant correlation between EQ5D-5L scores and oligomycin-induced changes in TMRM supports this hypothesis.

ATP synthase is known to be regulated by inhibitor factor 1 (IF1) with IF1 knock out models characterised by increases in ATP hydrolysis [36]. We speculate that in LC IF1 interaction with ATP synthase is impeded or perturbed, making IF1 a potential therapeutic target for LC. Moreover, ATP synthase perturbation might directly trigger an unwanted immune response, as the IF1/ATP synthesis coupling has been linked to the immune response [36,37].

Elevated ATP synthase-mediated ATP hydrolysis has also been observed in a mouse model of Duchenne Muscular Dystrophy. Notably, treatment with Epicatechin, a selective inhibitor of ATP hydrolysis that binds to complex V, was shown to increase ATP levels and improve muscle force without altering mitochondrial content [38]. This suggests that (+)-Epicatechin could be a potential treatment for Long COVID. More broadly, bioenergetic status could serve as a molecular marker to assess the efficacy of potential therapies, enabling the identification of drug-responsive versus non-responsive individuals and optimizing dosing strategies for a personalized medicine approach.

### Mitochondrial dysfunction is associated with clinical outcome in long COVID

The extent to which this apparent inefficient bioenergetic profile in PBMCs has a pathophysiological role in each of the individual clinical manifestations of long COVID is yet to be proven. Correlation analysis from this study suggests a possible association with fatigue and autonomic function but larger studies with more robust clinical assessments characterising multiple aspects of the LC syndrome including coagulation, cardiorespiratory reserve, measures of autonomic dysfunction together with viral profiling are required to confirm and ascertain the causal or consequential nature of this association. Conceivably, inefficient complex V function will result in diminished cytosolic ATP availability, particularly as no compensatory increase in glycolysis was observed. Heightened oxygen consumption rate derived from complex I–IV activity in context of aberrant complex V function will render the individual susceptible to the production of mitochondrial reactive oxygen species production. This may have the potential to further potentiate mitochondrial dysfunction resulting in a sustained pathological state. Compromised bioenergetic status coupled with a pro-inflammatory state is a clinical phenotype of Long COVID characterised by diminished organ reserve, in particular cardiopulmonary reserve [39].

Sexual dimorphism in mitochondrial phenotype was observed in LC cases with more pronounced increases in oxygen consumption rates in males and more striking differences in membrane potential in females. Estrogen pathways have been implicated in increases in mitochondrial content and components of the electron transport chain [40]. Testosterone pathways have been associated with an increase in complex V activity [41]. This may account for the observations in this study, but the literature base is sparse with regards to issues specificity of sex hormone effects on mitochondrial function and this interpretation is speculative.

BMI was observed to be significantly different between the LC and control groups yet was not found to be significantly correlated with any of the mitochondrial indices. This may be a function of study power or reflection of the imperfect nature of BMI as a measure of body composition and insulin resistance. BMI is not a direct measure of metabolically active visceral adipose tissue, an organ known to be associated with mitochondrial dysfunction [42,43]. Indeed, no participant in this study had a medical history of diabetes. An important consideration in future studies will be the interaction between PBMC subpopulations, metabolically active adipose tissue and insulin resistance and the extent to which the combined bioenergetic defects are synergistic. Ethnicity has also been identified as a risk factor for developing long COVID [44]. Whilst matching for ethnicity could not realistically be achieved within the confines of the study, there were no significant differences in ethnicity between LC and control groups.

### Current landscape of understanding of mitochondrial function in long COVID

This study adds to the existing literature base by describing a novel defect at the level of complex V. Previous studies have suggested an aberrant mitochondrial phenotype in LC but without specifically evaluating integrity of oxidative phosphorylation and glycolysis on live cells. Gunter et al. observed higher levels of tricarboxylates in the plasma of LC cases, indicating an altered fatty acid metabolism and dysfunctional mitochondrial-dependent lipid metabolism [45]. These findings our consistent with the findings from this study in which alterations in oxidative phosphorylation and complex V activity are described resulting in an increase in components of the Krebs cycle. Finnigan et al. conducted an early phase study targeting mitochondrial function in LC using AXA1125, an investigational product which has been shown to increase beta-oxidation and improve bioenergetics in preclinical studies [46]. The premise for the study was that the clinical syndrome of LC is suggestive of impaired bioenergetics. The authors observed an improvement in fatigue score but no significant difference in phosphocreatine recovery rate as an indirect marker of myocyte oxidative metabolism. This improvement in symptoms may reflect an improvement in non-myocyte mitochondrial dysfunction consistent with the findings in this study.

### Study limitations

Our study was designed as a pilot study and as such, has a number of limitations. Sample size was relatively small and whilst the groups were matched for age, there were differences between the groups with regards to BMI, ethnicity and male to female ratio with the potential to introduce confounding factors. Significant differences in BMI were observed between the groups although no significant correlation between BMI and oxygen consumption rate was observed in the case cohort. Lehrer et al. described a similar observation following Seahorse analysis of PBMCs in individuals with a mean BMI of 27, finding no significant correlation of BMI with any of the mitochondrial parameters assessed [47]. The small sample size did not allow for multiple comparisons and post hoc analysis to stratify by sex further reduced the statistical power. The study also did not further characterise the individual monocyte and lymphocyte subsets of the PBMC population and would be an important focus of future studies. Previous studies have observed reductions in mitochondrial DNA gene expression in CD8+ lymphocytes (including memory T cells) of cases with acute SARS–CoV-2 infection [29] and increased oxidative phosphorylation in the monocytes of cases with acute SARS–CoV-2 with distinct metabolic phenotypes in T cell subpopulations [48].

### Future directions

A larger, longitudinal, observational study would allow for the dynamic assessment of mitochondrial function. The data from this study suggests that randomisation should be stratified by sex given the different mitochondrial function profiles observed between female and male cases, consistent with previous observations that PBMC mitochondrial function is distinct between healthy control males and females [49]. PBMCs comprise a mixed population of B and T lymphocytes, monocytes, and NK cells. Further work is required to ascertain which populations may be responsible for the observed phenotypes in this study. Cell sorting methods could be employed in future studies to stratify these subpopulations prior to mitochondrial analysis. Future mechanistic studies could include further analysis of IF-1 including direct interactions with SARS–CoV-2 spike protein and characterization of AMPK and TORC intracellular signaling pathways. Mitochondrial-specific fluorescent temperature indicators may be used to quantify organelle-specific heat production [50].

Future studies should also explore whether the discordant transcriptomic profile of nuclear and mitochondrial DNA genomes observed in acute SARS–CoV-2 infection is a feature of long COVID mitochondrial dysfunction. In acute SARS–CoV-2 infection, a discordant expression in mitochondrial and nuclear-encoded oxidative phosphorylation genes is observed in a number of tissues including CD8^+^ T cells from bronchiolar lavage fluid and PBMCs [45]. In conjunction with measures of cell-mediated immunity, serological markers of inflammation should be characterised to determine cytokine and chemokine profiling particularly given that insulin resistance is an important predisposing factor to complications of SARS–CoV-2 infection and potentially create a proinflammatory milieu to potentiate the pathophysiological effects of PBMCs identified in this study.

In conclusion, this study identifies mitochondrial function abnormalities in the PBMCs of LC cases which are distinct from the mitochondrial phenotype of acute SARS–CoV-2 infection. Our findings are consistent with the hypothesis that there is an abnormality of complex V function which is evidence of bioenergetic inefficiency. The results suggest that PBMC mitochondrial function might be a future biomarker of LC. Future appropriately powered and ideally longitudinal observational studies are required to validate these findings and determine the degree to which these bioenergetic changes are imprinted irreversibly or determinants of recovery.

## Supplementary Material

Supplemental Material

## Data Availability

The anonymised data that support the findings of this study are available from the corresponding author, JM, upon reasonable request.
