# Supplementary material for: Mitochondrial function is impaired in long COVID patients
Source: Ann Med. 2025 Aug 12;57(1):2528167. doi: 10.1080/07853890.2025.2528167 (PMC12344680; doi:10.1080/07853890.2025.2528167)

**Supplementary Data**

**Supplementary Table 1**

| Mitochondrial Assay | Healthy Control (Median (Q1-Q3)) | | | Long COVID (Median (Q1-Q3)) | | | P value (Mann Whitney) | | | | |
| --- | --- | --- | --- | --- | --- | --- | --- | --- | --- | --- | --- |
|  | All | Female | Male | All | Female | Male | HC-A vs LC-A | HC-F vs LC-F | HC-M vs LC-M | HC-F vs HC-M | LC-F vs LC-M |
| Basal OCR | 89.77  (83.40-96.24) | 94.88  (87.47-100.40) | 86.72  (69.80-92.95) | 116.10  (99.20-147.30) | 116.00  (94.15-151.10) | 143.50  (105.90-146.50) | **<0.0001** | **0.0176** | **0.0003** | 0.0650 | 0.5332 |
| ATP-linked OCR | 86.24  (73.81-91.15) | 90.81  (78.79-93.44) | 82.38  (64.75-88.24) | 113.80 (98.63-142.40) | 113.10 (87.79-140.50) | 134.50  (101.20-142.40) | **<0.0001** | **0.0254** | **0.0003** | 0.0830 | 0.4980 |
| Proton-leak | 6.02  (4.15-9.73) | 6.75  (2.47-10.53) | 5.71  (4.15-7.83) | 5.69  (0.19-11.89) | 3.67  (0.17-10.34) | 7.32  (2.27-13.74) | 0.7001 | 0.5002 | 0.6126 | 0.7209 | 0.3138 |
| ECR | 28.59  (23.98-40.73) | 27.06  (23.98-35.68) | 30.40  (22.63-44.90) | 31.25  (27.01-45.76) | 31.48  (28.34-45.96) | 27.10  (26.90-32.15) | 0.4042 | 0.2519 | 0.6943 | 0.7209 | 0.3998 |
| Net TMRM | 0.09  (0.05-0.12) | 0.05  (0.04-0.09) | 0.11  (0.09-0.16) | 0.11  (0.07-0.22) | 0.12  (0.08-0.21) | 0.09  (0.07-0.38) | 0.0643 | **0.0032** | 0.7789 | 0.0148 | 0.8500 |
| Oligo-δTMRM | 37.91  (12.16-66.28) | 24.94  (10.60-75.40) | 42.26  (21.80-66.10) | -8.65  (-24.63- -2.46) | -5.90  (-15.59- 10.42) | -28.11  (-37.44- -6.58) | **<0.0001** | **0.0002** | **0.0059** | 0.7984 | **0.0190** |
| FCCP- δTMRM | -30.44  (-38.42- -23.48) | -25.31  (-36.05- 18.48) | -34.48  (-42.72- -26.96) | -46.51  (-54.61- -23.47) | -46.53  (-54.11- -29.52) | -29.86  (-58.22- -24.10) | 0.0503 | **0.0362** | 0.8665 | 0.2345 | 0.6850 |
| Ant/Rot-δTMRM | -35.26  (-50.50- -32.53) | -31.80  (-35.88- -21.53) | -43.78  (-52.66- -29.70) | -45.39  (-55.08- -32.53) | -48.38  (-54.76- -36.41) | -35.35  (-65.20- -23.22) | 0.0710 | **0.0096** | 0.7789 | 0.0830 | 0.4640 |

**Supplementary Table 2**

|  | Long COVID (Total) | | Long COVID (Female) | | Long COVID (Male) | |
| --- | --- | --- | --- | --- | --- | --- |
| Clinical Parameter | Median (Q1-Q3) | n | Median (Q1-Q3) | n | Median (Q1-Q3) | n |
| Fatigue Assessment Scale | 34.00 (31.00-39.00) | 23 | 35 (31-39) | 18 | 31.00 (30.00-39.00) | 5 |
| MRC Dyspnoea scale | 2.00 (1.00-3.00) | 24 | 2 (1-3) | 18 | 2.00 (1.00-3.00) | 6 |
| Resting HR/Predicted Maximum HR (%) | 45.00 (39.25-48.75) | 24 | 45 (39-48) | 19 | 47.49 (41.13-53.10) | 5 |
| EQ5D-5L | 11.00 (9.00-13.00) | 23 | 11 (8.5-13) | 17 | 11.00 (8.50-16.75) | 6 |
| Body Mass Index | 25.60 (32.51-22.80) | 27 | 24.55 (22.60-32.27) | 20 | 27.00 (25.60-32.90) | 7 |
| Myalgia | n (%) | 27 | n (%) | 20 | n (%) | 7 |
| Yes | 13 (48) |  | 8 (40%) |  | 5 (71%) |  |
| No | 14 (52) |  | 12 (60%) |  | 2 (29%) |  |

**Supplementary Table 3**

|  | Healthy Control | Long COVID |
| --- | --- | --- |
| n | 16 | 27 |
| ***Oxygen Consumption Rate*** |  |  |
| Median | 89.77 | 116.10 |
| 25% Percentile | 83.40 | 99.20 |
| 75% Percentile | 96.24 | 147.30 |
|  |  |  |
| ***ATP-Oxygen Consumption Rate*** |  |  |
| Median | 86.24 | 113.80 |
| 25% Percentile | 73.81 | 98.63 |
| 75% Percentile | 91.15 | 142.40 |
|  |  |  |
| ***Proton Leak*** |  |  |
| Median | 6.02 | 5.69 |
| 25% Percentile | 4.15 | 0.19 |
| 75% Percentile | 9.73 | 11.89 |
|  |  |  |
| ***ECAR*** |  |  |
| Median | 28.59 | 31.25 |
| 25% Percentile | 23.98 | 27.01 |
| 75% Percentile | 40.73 | 45.76 |
|  |  |  |
| ***TMRM*** |  |  |
| Median | 0.09 | 0.11 |
| 25% Percentile | 0.05 | 0.07 |
| 75% Percentile | 0.12 | 0.22 |
|  |  |  |
| ***ψM*** |  |  |
| Median | 0.05 | 0.06 |
| 25% Percentile | 0.04 | 0.04 |
| 75% Percentile | 0.06 | 0.11 |
|  |  |  |
| ***%CS*** |  |  |
| Median | 1.83 | 2.21 |
| 25% Percentile | 1.25 | 1.49 |
| 75% Percentile | 2.38 | 2.65 |
|  |  |  |
| ***TOM20*** |  |  |
| Median | 10.17 | 12.09 |
| 25% Percentile | 8.96 | 8.61 |
| 75% Percentile | 12.55 | 14.38 |

**Supplementary Figure 1**

Comparison of mitochondrial parameters of PBMC from patients (n=27) and controls (n=16). A illustrates the differences in mitochondrial against genomic DNA ratio between controls (n=16, white box) and patients (n=27, grey boxes). (Data is expressed as median with interquartile range, and medians are compared using the MannWhitney U test). The scattered plot (B) is used to illustrate the difference between the mtDNA rearrangement detected.


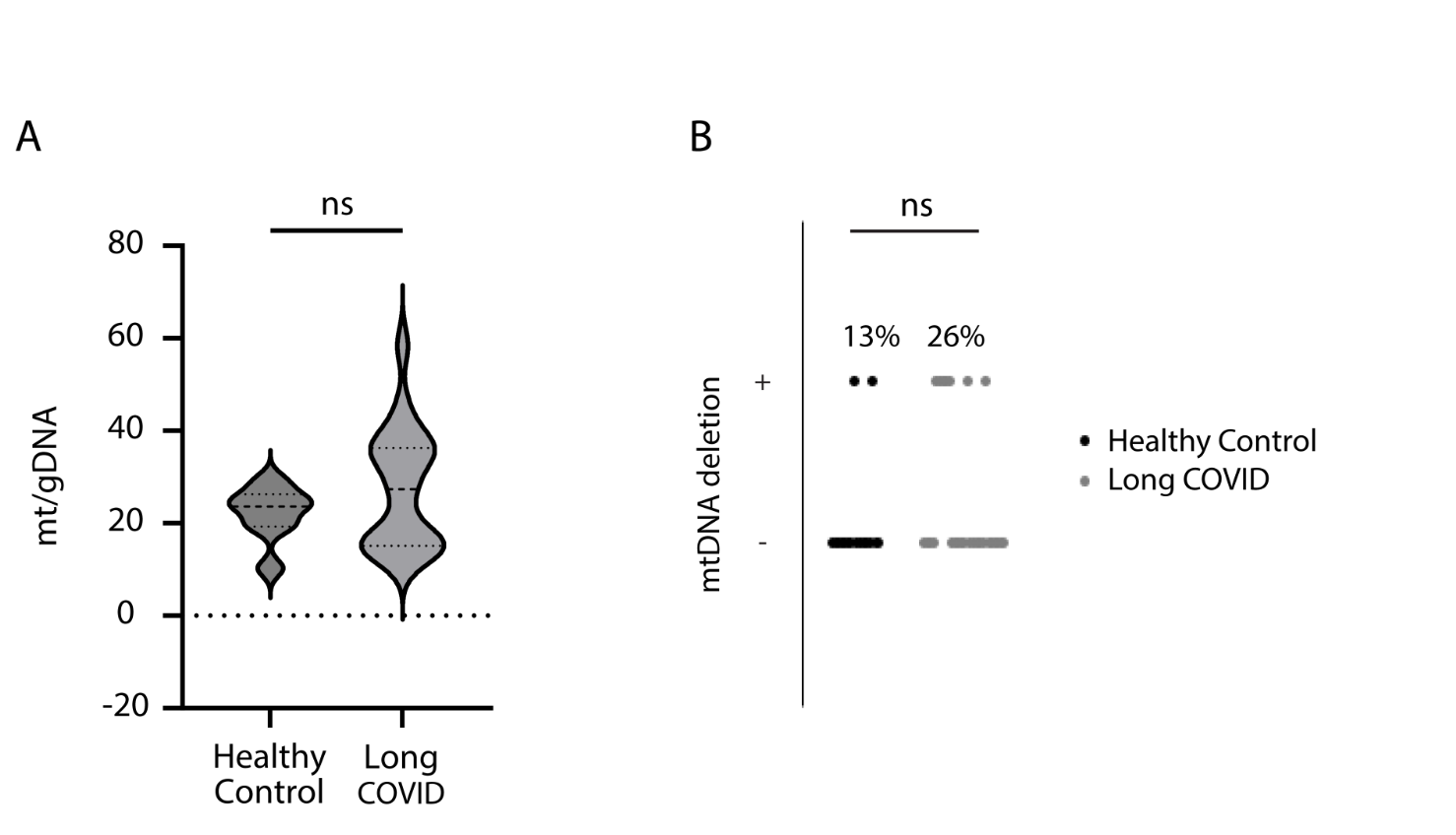

Supplement: Supplemental Material [file IANN_A_2528167_SM0552.docx]
